# Supplementary material for: Proteomics of Mouse Heart Ventricles Reveals Mitochondria and Metabolism as Major Targets of a Post-Infarction Short-Acting GLP1Ra-Therapy
Source: Int J Mol Sci. 2021 Aug 13;22(16):8711. doi: 10.3390/ijms22168711 (PMC8395861; doi:10.3390/ijms22168711)
Supplement: Supplementary file 1 [file ijms-22-08711-s001.zip › Supplementary Figures.pdf]

Figure S1: Heatmap of top 20 significantly DE proteins in the intragroup comparison

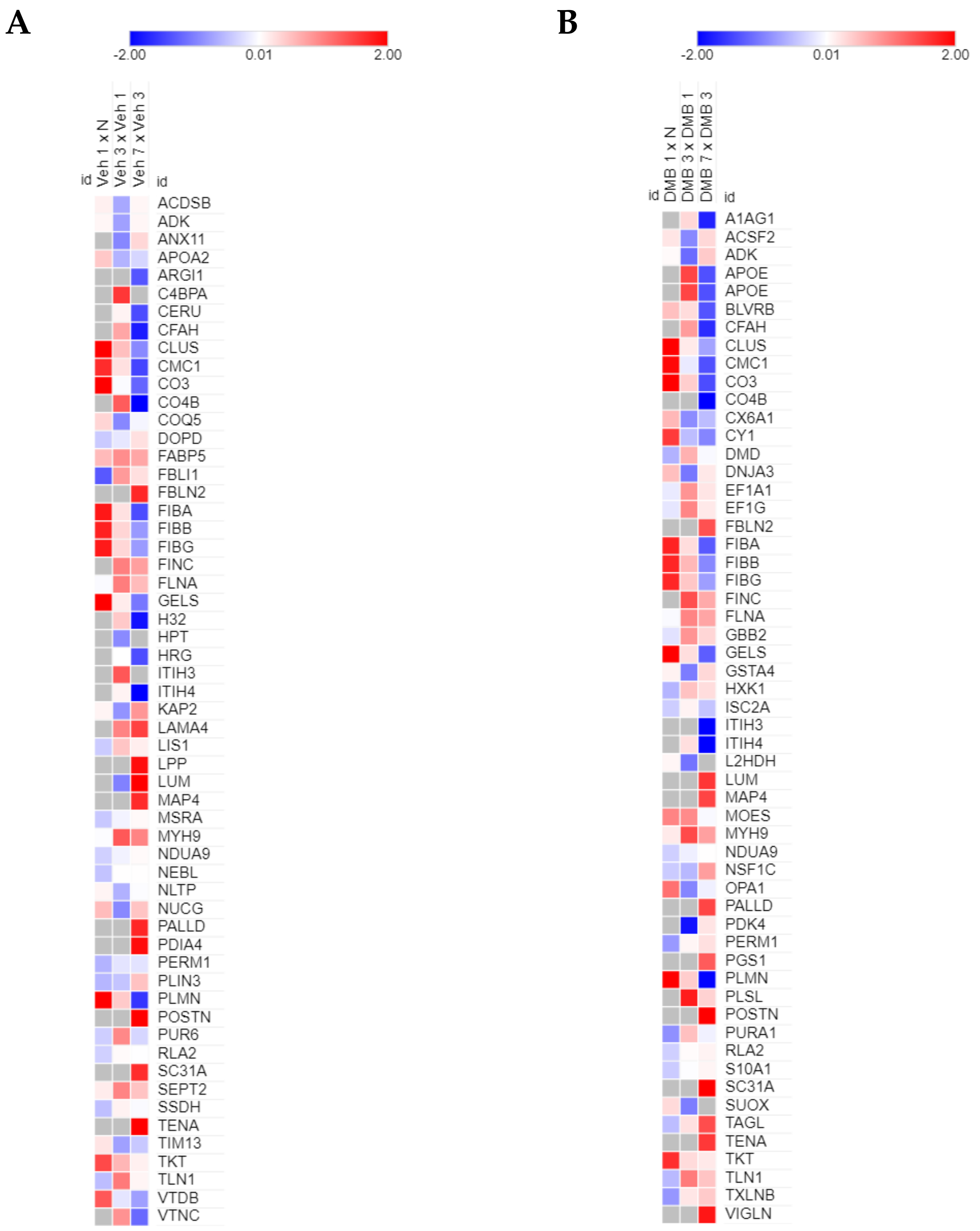

Figure S1. Heatmap of the top 20 significant DEPs in the intragroup comparison. A comparative analysis was done between each time point and its preceding time point intragroup (vehicle or DMB). Differentially expressed proteins (DEPs; $p < 0.05$) were identified and the log2 fold-change values from the top 10 downregulated and upregulated proteins at each time point were used to obtain the heatmap. (**A**) Heatmap of the top 20 DEPs at each time point in the vehicle group; (**B**) heatmap of the top 20 DEPs at each time point in the DMB group.

Figure S2. Top 30 biological processes of the unique proteins and DEPs in the intragroup comparison

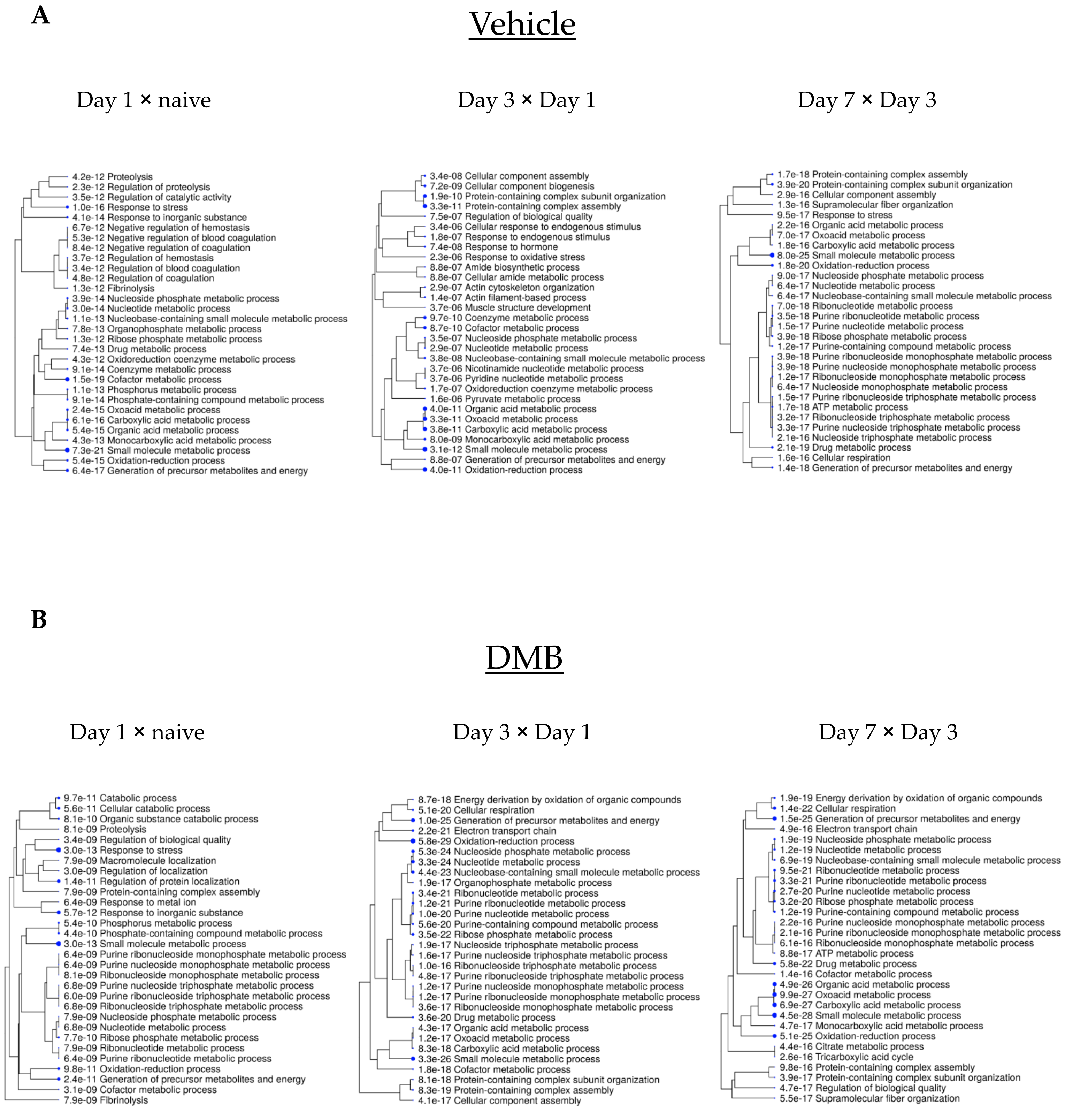

Figure S2. Top 30 biological processes of the unique proteins and DEPs in the intragroup comparison. The expression of proteins from each time point was compared to its preceding time point intragroup to identify unique proteins and DEPs ($p < 0.05$). A list containing all unique proteins and DEPs at each time point was uploaded on ShinyGO v0.61 and biological processes were obtained. Biological processes were clustered according to the amount of proteins they have in common, and the size of the blue dots represent *p*-values (the bigger the size, the lower the *p*-value). (**A**) Biological processes of unique proteins and DEPs in the vehicle-treated group; (**B**) biological processes of unique proteins and DEPs in the DMB-treated group.

Figure S3. ShinyGO analysis of the top 30 biological processes of the unique and DEPs between DMB vs vehicle at each time point

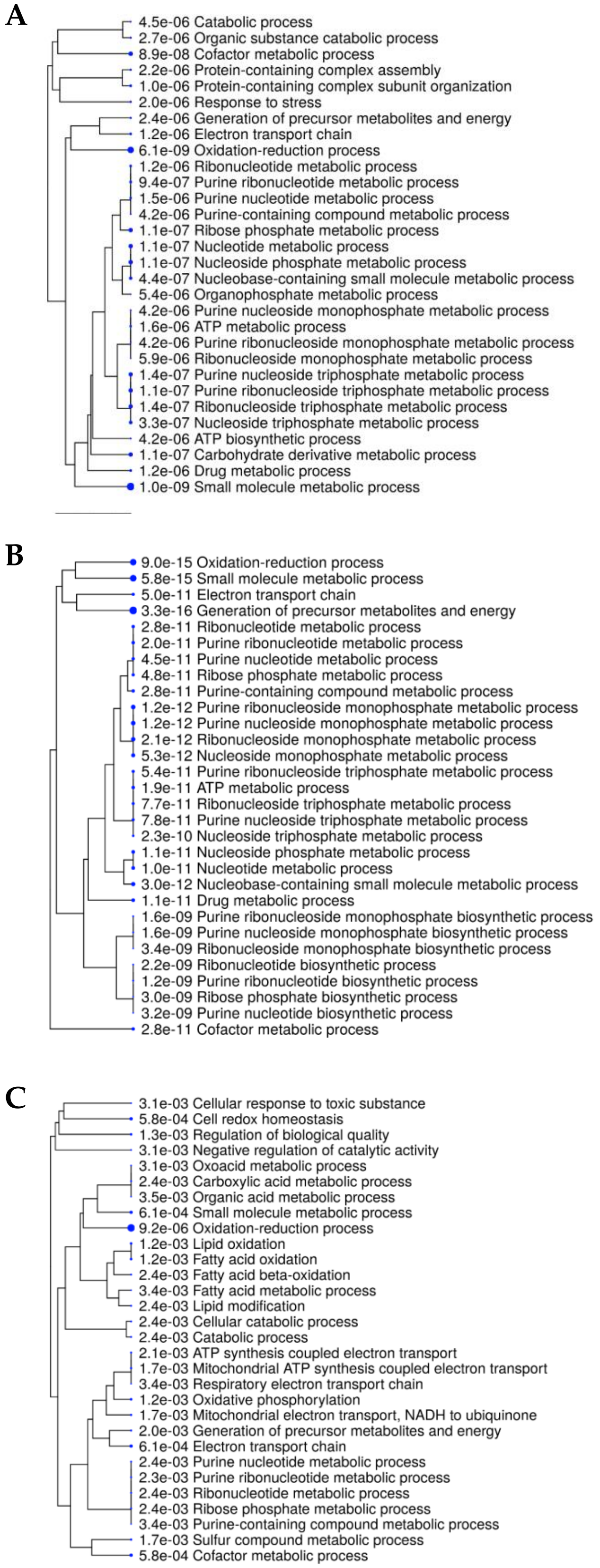

Figure S3. ShinyGO analysis of the top 30 biological processes of the unique proteins and DEPs between DMB vs. vehicle at each time point (1, 3 or 7 days after PCAL) to identify unique proteins and DEPs ($p < 0.05$). A list containing all unique proteins and DEPs at each time point was uploaded on ShinyGO v0.61 and biological processes were obtained. Biological processes were clustered according to the amount of proteins they have in common, and the size of the blue dots represent p-values (the bigger the size, the lower the p-value). (**A**) Biological processes modulated by DMB on day 1 post-PCAL; (**B**) biological processes modulated by DMB on day 3 post-PCAL; (**C**) biological processes modulated by DMB on day 7 post-PCAL.

Figure S4. ClueGO analysis pie-charts of the biological processes identified as differentially modulated by DMB in the first week post-PCAL in mice

A

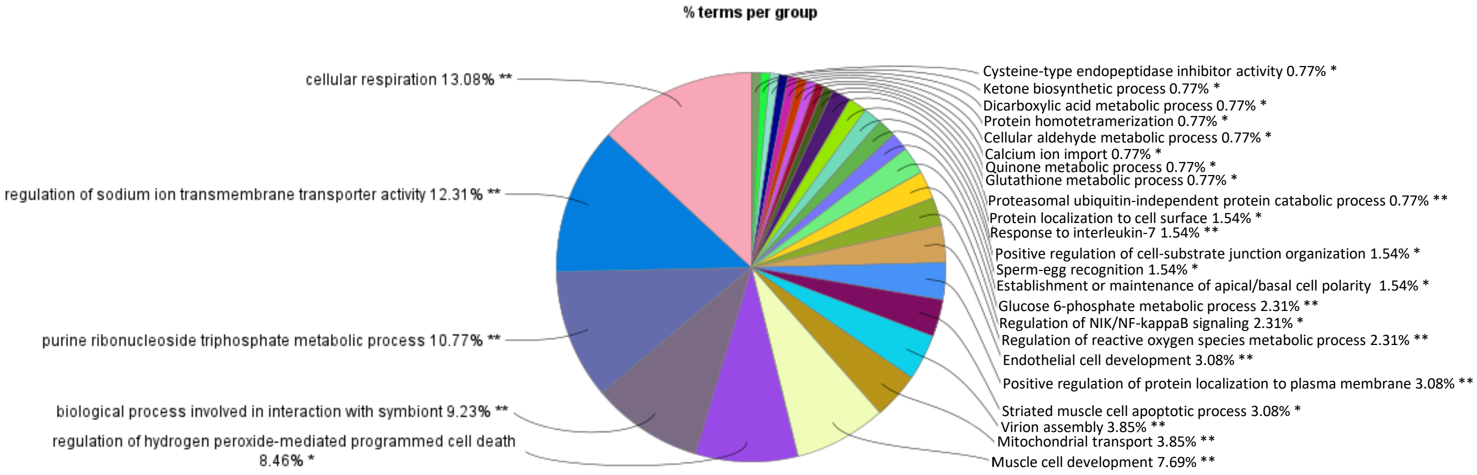

B

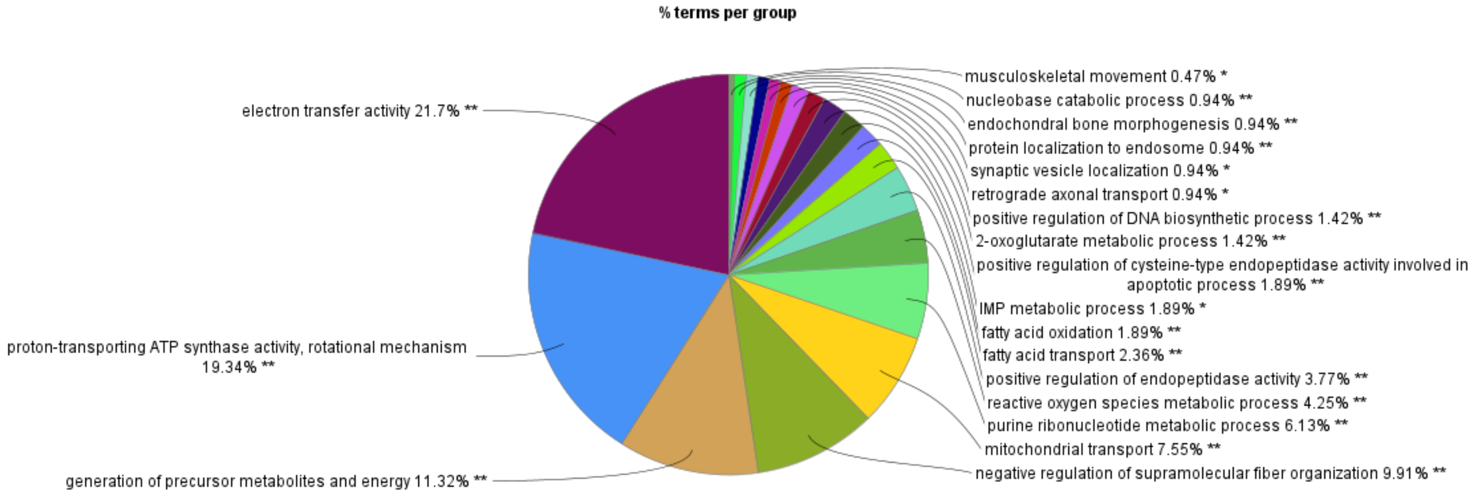

C

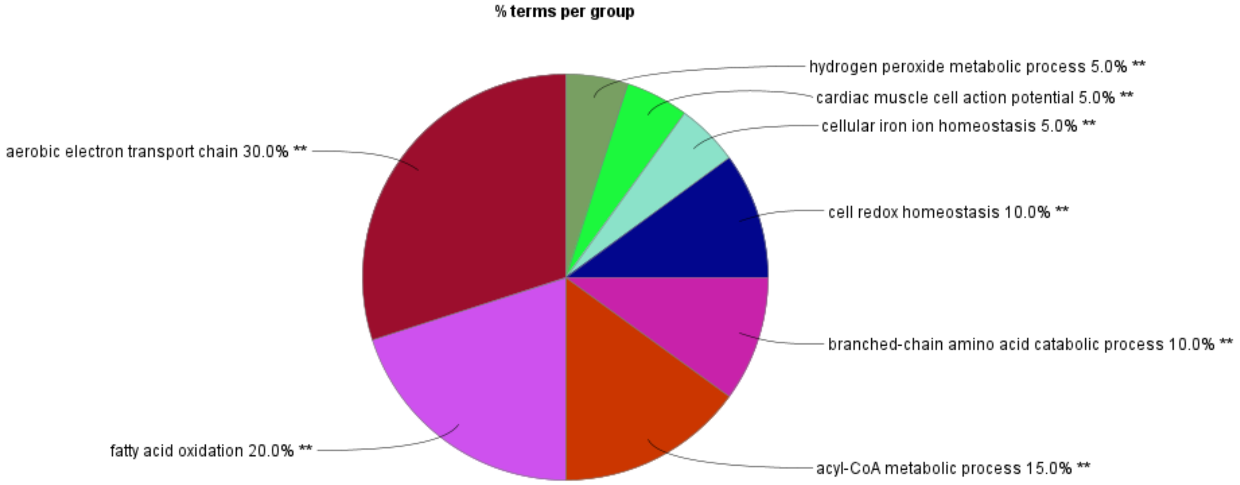

Figure S4. ClueGO analysis pie-charts of the biological processes identified as differentially modulated by DMB in the first week post-PCAL in mice. A cross-comparative analysis was done at each time point (1, 3 or 7 days after PCAL) to identify unique proteins and DEPs ($p < 0.05$). A list containing all unique proteins and DEPs at each time point was uploaded on ClueGO and biological processes were obtained. (**A**) Total biological processes identified by ClueGO on day 1 post-PCAL; (**B**) total biological processes identified by ClueGO on day 3 post-PCAL; (**C**) total biological processes identified by ClueGO on day 7 post-PCAL. *$p < 0.05$; **$p < 0.001$
