# Supplementary material for: Proteomics of Mouse Heart Ventricles Reveals Mitochondria and Metabolism as Major Targets of a Post-Infarction Short-Acting GLP1Ra-Therapy
Source: Int J Mol Sci. 2021 Aug 13;22(16):8711. doi: 10.3390/ijms22168711 (PMC8395861; doi:10.3390/ijms22168711)
Supplement: Supplementary file 1 [file ijms-22-08711-s001.zip › Supplementary Table S1.pdf]

Table S1. Differentially expressed (DE) mitochondrial proteins throughout the course of early remodeling

Vehicle	Upregulated	Downregulated	DMB	Upregulated	Downregulated
Day 1 vs Naive	AIFM1, CATB, CLH1, CLUS, CMC1, COX5B, CX6A2, CY1, GBLP, GRP78, MIC60, ODPB, PDIA3, QCR8 SAP	ABHDB, ACADL, ACON, ACOT2, ACS2L, ACSL1, ADCK3, ALDOA, ATP5J, BCAT2, CISY, CLYBL, CYC, ETFA, ETFB, FIS1, IDHP, ISC2A, LDHA, LDHB, MDHC, MSRA, MUTA, MYH6, NDUA9, NDUV2, ODP2, ODPB, SCOT1, SSDH, SUCA, THIM UBA1	Day 1 vs Naive	AMPL, ATPG, CATB, CLH1, CLUS, CMC1, COF1, COX5A, CY1, DNJA3, GBLP, GRP78, KAD3, MIC19, MIC60, MSRB2, OPA1, PDIA3 VDAC1	AL4A1, DMD, ETFB, HXK1, HXK2, IDHP, ISC2A, LDHA, MYH6, NDUA9, ODP2, UBA1
Day 3 vs Day 1	CALM, CATB, CLH1, CLIC4 , COF1, CRYAB, CX7A1, FIS1, HNRPK, KPYM, LDHA, NDUA4, TGM2	ACADV, ACDSB, ACO13, ACPM, ACSL1, ADCK3, AIFM1, CACP, COQ5, COX5B, ECHA, ECI2, ES1, ETHE1, HXK2, KCRS, LDHB, LONM, MMSA, MUTA, NDUBA, NDUS7, NLTP, NNRE, NUCG, ODP2, PCCB, PDK2, PRDX5, SUCB1, SYIM, THIL, TIM13, VWA8	Day 3 vs Day 1	1433Z, ANXA1, CATB, CATD, CLH1, CLIC1, CLIC4, COF1, CRYAB, DMD, GRP78, HSPB7, PDIA3, SAP, THIO, TRAP1	AATM, ACADL, ACADM, ACADS, ACADV, ACD10, ACDSB, ACON, ACSF2, ADCK3, AMPL, AT5F1, ATP5H, ATPA, ATPB, ATPD, CH60, CLYBL, COX41, COX5A, COX5B, CX6A1, CX6B1, CY1, CYC, DCMC, DHPR, DHRS4, DLDH, DNJA3, ECH1, EFTU, ETFA, ETFB, ETFD, ETHE1, FUMH, GRP75, GRPE1, GSTA4, HCD2, IDH3A, IDHC, ISCA2, IVD, KAD3, KAT3, KCRS, L2HDH, LDHB, LONM, MCEE, MDHC, MDHM, MIC13, MIC19, MIC60, MYH6, NDUA5, NDUAB, NDUBA, NDUBB, NDUS1, NDUS3, NDUS6, NDUV2, ODO1, ODO2, ODPA, ODPX, OPA1, PDK2, PDK4, PFD2, PHB, PHB2, PRDX3, PRDX5, PTRF, QCR1, SCOT1, SDHA, SDHB, SODM, SUCA, SUOX, TACO1, THIL, THIM, THIOM, TIM13, TIM44, UCRI, VDAC1, VDAC3, VWA8
Day 7 vs Day 3	ANXA1, CATB, CATD, CLH1, CLIC1, DNM1L, DPYL2, G3P, HXK1, LEG3, MUTA, PDIA3, PUR9, SAP, UBA1	AATM, ACADL, ACADM, ACADS, ACADV, ACD10, ACON, ACOT2, ACOX1, ACPM, ARGI1, ATP5H, ATPB, ATPD, ATPG, ATPO, CATA, CH60, CLUS, CLYBL, CMC1, COQ3, CRYAB, CY1, DCMC, DECR, DHB8, DHRS4, DLDH, ECH1, ECHA, ECI1, EFTU, ETFA, FIS1, FUMH, GSTK1, IDH3A, IVD, KAD3, LPPRC, MACD1, MCCA, MDHM, MIC19, MIC60, MTX2, NDUA4, NDUA5, NDUAA, NDUF3, NDUS1, NDUS2, NDUS3, NDUS6, NDUV2, NDUV3, NFU1, NIPS2, ODO1, ODO2, ODPB, ODPX, PCCA, PFD2, PHB, PHB2, PPIF, PRDX2, PRDX3, QCR1, QCR2, QCR7, QCR8, SCOT1, SDHA, SODC, SUCB1, THIM, VDAC1	Day 7 vs Day 3	ANXA1, BDH, CATB, CATD, CLH1, COF1, DNM1L, G3P, GLOD4, MDHC, MYH6, PDIA3, SAP, SND1, SYVC, TGM2	ACADM, ACADV, ACON, ACOT2, ACPM, ACS2L, ACSL1, ACYP2, ADT1, ATPG, C1QBP, CLUS, CMC1, COQ5, COQ7, COQ9, COX5A, COX5B, CX6A2, CY1, DECR, DHB8, DLDH, ECH1, ECHA, ECHB, ECHM, ECI1, EFTU, ES1, ETFA, ETFB, FIS1, FUMH, HCDH, HIBCH, HINT2, HMGCL, IDH3A, IDHP, ISC2A, KAD4, MCCB, MDHM, MIC60, MMSA, MTX2, NDUAA, NDUAC, NDUS1, NDUS3, NDUS4, NDUV2, NDUV3, NIPS2, NIT2, ODB2, ODO1, ODO2, ODPA, ODPB, PCCA, PCCB, PHB, PHB2, PRDX3, QCR1, QCR6, SDHA, SODM, SUCA, SUCB1, THIL, THIM, VDAC1, VDAC3, VWA8
